# Countervailing effects of income, air pollution, smoking, and obesity on aging and life expectancy: population-based study of U.S. Counties

**DOI:** 10.1186/s12940-016-0168-2

**Published:** 2016-08-12

**Authors:** Ryan T. Allen, Nicholas M. Hales, Andrea Baccarelli, Michael Jerrett, Majid Ezzati, Douglas W. Dockery, C. Arden Pope

**Affiliations:** 1 Department of Economics, Brigham Young University, 142 FOB, Provo, UT 84602 USA; 2 Department of Environmental Health, Harvard T.H. Chan School of Public Health, 665 Huntington Ave, Boston, MA 02115 USA; 3 UCLA Fielding School of Public Health, 650Charles E. Young Drive South, 56-070B CHS, Los Angeles, California 90095 UK; 4 MRC-PHE Centre for Environment and Health, School of Public Health, Imperial College London, London, UK

**Keywords:** Air pollution, Life expectancy, Income, Smoking, Obesity, Economic tradeoffs

## Abstract

**Background:**

Income, air pollution, obesity, and smoking are primary factors associated with human health and longevity in population-based studies. These four factors may have countervailing impacts on longevity. This analysis investigates longevity trade-offs between air pollution and income, and explores how relative effects of income and air pollution on human longevity are potentially influenced by accounting for smoking and obesity.

**Methods:**

County-level data from 2,996 U.S. counties were analyzed in a cross-sectional analysis to investigate relationships between longevity and the four factors of interest: air pollution (mean 1999–2008 PM_2.5_), median income, smoking, and obesity. Two longevity measures were used: life expectancy (LE) and an exceptional aging (EA) index. Linear regression, generalized additive regression models, and bivariate thin-plate smoothing splines were used to estimate the benefits of living in counties with higher incomes or lower PM_2.5_. Models were estimated with and without controls for smoking, obesity, and other factors.

**Results:**

Models which account for smoking and obesity result in substantially smaller estimates of the effects of income and pollution on longevity. Linear regression models without these two variables estimate that a $1,000 increase in median income (1 μg/m^3^ decrease in PM_2.5_) corresponds to a 27.39 (33.68) increase in EA and a 0.14 (0.12) increase in LE, whereas models that control for smoking and obesity estimate only a 12.32 (20.22) increase in EA and a 0.07 (0.05) increase in LE. Nonlinear models and thin-plate smoothing splines also illustrate that, at higher levels of income, the relative benefits of the income-pollution tradeoff changed—the benefit of higher incomes diminished relative to the benefit of lower air pollution exposure.

**Conclusions:**

Higher incomes and lower levels of air pollution both correspond with increased human longevity. Adjusting for smoking and obesity reduces estimates of the benefits of higher income and lower air pollution exposure. This adjustment also alters the tradeoff between income and pollution: increases in income become less beneficial relative to a fixed reduction in air pollution—especially at higher levels of income.

## Background

Relationships between longevity and income, air pollution, obesity, and smoking are well documented. Specifically, the positive relationship between income and health is the focus of much discussion, often related to difficulties of making causal interpretations of observed associations [1]. It is obvious that the mere presence of dollars and cents in a bank account does not lead to improved longevity. Rather, research focuses on the mechanisms and correlations by which higher incomes might improve longevity [2, 3]. Some research suggests that higher incomes lead to increased access to medical care [4, 5]. Other studies provide evidence that income is correlated to other traits or characteristics that lead to improved health [6]. For example, higher incomes have been found to be correlated to behaviors that influence health, such as propensity to gain weight and smoking habits [7, 8]. Thus research evaluating associations between income and longevity unavoidably involves underlying correlates of income.

Relative to income, other factors may have more direct biological relationships to longevity. There is extensive evidence demonstrating that exposure to air pollution has adverse health effects and increases the risk of pulmonary and cardiovascular morbidity and mortality [9–11], thereby leading to decreases in population longevity [12]. Additionally, the link between smoking and reduced longevity has been well established [13, 14], as has the link between obesity and reduced life expectancy [15, 16].

Public health research seeks to identify (and ideally improve) the factors that influence longevity. With this goal in mind some questions follow naturally: What is the most effective way to improve longevity? Of the factors discussed above, is any one factor crucially more important than the others? Additionally, questions arise about tradeoffs. In the present study, the term “tradeoff” refers to how changes in one factor influence longevity relative to changes in another factor. Quantifying such tradeoffs is important if changes in one factor are accompanied by changes in another factor. For example, tradeoffs between pollution and income might be particularly important if achieving a higher median income came at the expense of higher levels of pollution, a phenomenon which could possibly be explained by policies which promote economic growth at the environment’s expense. Another example of this may be air pollution and obesity: some studies have found that increases in air pollution are correlated with increases in obesity [17–19]. Ideally, a policy goal to improve longevity would simultaneously increase incomes, reduce smoking, lower obesity, and limit air pollution exposure. Limited resources and inextricable relationships between factors may make such a policy unlikely. Therefore, policy makers must understand tradeoffs between these factors in order to improve longevity in the most efficient and impactful way.

Previous studies have examined tradeoffs between these factors, with trends in some factors possibly counteracting trends in others. For example, there is some evidence indicating that the improvements in longevity from decreased smoking are being offset by increases in obesity prevalence [20]. Researchers have also investigated the relative effectiveness of changes in income versus changes in pollution [21, 22]. Such interactions are complex and require additional research.

The objective of this study was to evaluate longevity tradeoffs between income and pollution using population-based county-level data and multiple measures of longevity, including life expectancy (LE) and an index of exceptional aging (EA). The study evaluates the longevity benefits of higher income relative to lower air pollution and illustrates how the relative effectiveness of income is affected by its correlation with smoking and obesity.

## Methods

This study used a cross-sectional study design applied to county-level data in order to analyze how EA and LE are associated with income, air pollution, smoking, and obesity. Linear regression, generalized additive models, and bivariate thin-plate spline smoothers were used in order to analyze these associations.

### EA and LE data

An index of county-level EA was calculated for 2,996 counties in the United States using publicly available census data [23]. The EA index is defined as the number of 85–94 year olds in 2010 divided by the number of 55–64 year olds in 1980, multiplied by 10,000. Details concerning this measure of EA are documented elsewhere [24]. Additionally, previously reported [25] estimated county-level male and female LE from 2002 to 2007 were averaged to obtain mean county-level LE.

### Demographic and income data

County-level measurements of median age, median income, percent black, percent Hispanic, and the percent of the population over age 65 were obtained from the U.S. Census [23]. Because the EA index is calculated from population counts, it was essential to control for the migration of elderly individuals. Thus age-specific migration rates were obtained for the decades of the 1980s, 1990s, and 2000s [26]. These rates are calculated by taking net migration (observed final population minus expected final population) over a given decade and dividing it by the expected population at the end of the decade, and then multiplying the results by 100.

### Air pollution, smoking, and obesity data

Monthly fine particulate matter air pollution (PM_2.5_) estimates for each county were obtained for the period from 1999 to 2008 using traffic indicators, land-use regression, and Bayesian Maximum Entropy interpolation of land-use regression space-time residuals as documented elsewhere [27]. Pollution exposures were then assigned to be the average of these monthly estimates across this entire 10-year period. Counties from Hawaii and Alaska were not included in the analysis because of inadequate PM_2.5_ data. County-level estimates of percentage of adults who smoked daily in 2000 were obtained from the Institute for Health Metrics and Evaluation [28], and estimates of average obesity prevalence from 2004 to 2010 were obtained from the Centers for Disease Control and Prevention [29].

### Statistical analysis

Population weighted linear regression (weighting by the square root of 2000 population) was used to estimate the impacts of various factors on EA. The full model included income, pollution, obesity, and smoking, as well as percent black, percent Hispanic, median age, percent of individuals over age 65, and indicator variables for the nine census divisions. Age-specific migration rates for the relevant population were included to appropriately control for migration. An unadjusted model which excluded smoking and obesity was also estimated. Counties with extreme migration patterns (37 outlier counties as defined elsewhere [24]), were excluded from the analysis. Full and unadjusted linear regression models were also estimated using only counties with median incomes greater than $40,000 in order to investigate relationships at high levels of income.

Non-linear relationships between EA and covariates were examined using generalized additive models (GAM) with spline smooth functions for all covariates except the census division indicators. Similar to the linear regression models, GAM models were estimated using a full set of covariates that included smoking and obesity (full models) as well as without controls for smoking and obesity (unadjusted models). To explicitly examine how tradeoffs between PM_2.5_ and income affect EA, bivariate thin-plate smoothing splines of EA relative to both median income and PM_2.5_ were estimated. This method illustrates the combined non-linear impacts of median income and PM_2.5_ on a three-dimensional surface. Both a full model and an unadjusted model were estimated.

Comparable weighted linear regressions, generalized additive models, and thin-plate smoothing splines were also estimated using county-level LE in place of EA (weighting by the inverse of LE confidence intervals instead of by the square root of 2000 population). The GAM models and thin-plate smoothing splines were estimated using the R software [30] MGCV package by including “gam function” with penalized regression smoothers allowing for up to 4 degrees of freedom.

## Results

Table 1 presents summary statistics and data sources for all variables used in the analysis. On average, of 10,000 individuals aged 55–64 in a given county in 1980, about 2,276 survived to ages 85–94 in 2010. Average LE across all counties in the analysis was 76.9 years. Average PM_2.5_ exposure was 10.4 μg/m^3^, and average smoking and obesity rates were 21.6 % and 28.1 % respectively. Average median income across all counties in the analysis was approximately $35,100.

**Table 1:** Summary statistics and data sources

Variable (Units)	Mean (SD)	Source
EA^†^ (Index)	2275.8 (696.3)	U.S. Census 1980, 2010
LE^††^ (Years)	76.9 (2.0)	Kulkarni et al. 2011
PM_2.5_ (μg/m^3^)	10.4 (2.8)	Beckerman et al. 2013
Median Income ($1000s)	35.1 (8.6)	US Census 2000
Daily Smokers (%)	21.6 (3.7)	Institute for Health Metrics and Evaluation 2014
Obesity Prevalence (%)	28.1 (3.6)	Centers for Disease Control and Prevention 2013
Black (%)	8.7 (14.5)	US Census 2000
Hispanic (%)	6.2 (12.0)	US Census 2000
Median Age (Years)	37.4 (3.9)	US Census 2000
Over 65 (%)	14.8 (4.0)	US Census 2000
Migration Rates		
1980s, 55–60-year-olds^‡^	4.3 (16.6)	Winkler et al. 2013
1980s, 60–64-year-olds^‡^	7.0 (19.7)	Winkler et al. 2013
1990s, 65–70-year-olds^‡^	9.9 (19.9)	Winkler et al. 2013
1990s, 70–74-year-olds^‡^	4.0 (12.7)	Winkler et al. 2013
2000s, 75+ year-olds^‡^	−1.0 (14.8)	Winkler et al. 2013

† Exceptional Aging

†† Life Expectancy

‡ Age-specific migration rates were calculated by the net migration over the given decade divided by the expected population at the end of the decade, times 100, where net migration is the observed final population minus the expected final population

Table 2 presents weighted Pearson correlation coefficients for variables of interest. The two measures of longevity, EA and LE, were correlated (*r* = 0.51). PM_2.5_, median income, smoking, and obesity were all correlated with both measures of longevity. The correlation between EA and PM_2.5_ (*r* = −0.22) was weaker than correlations between EA and income, smoking, and obesity (*r* = 0.38, *r* = −0.32, and *r* = −0.41, respectively). Similarly, the correlation between LE and PM_2.5_ (*r* = −0.27) was also weaker than correlations between LE and income, smoking, and obesity (*r* = 0.68, *r* = −0.63, and *r* = −0.77, respectively). Additionally, median income was negatively correlated with smoking and obesity (*r* = −0.54 and *r* = −0.58, respectively), and weakly correlated with PM_2.5_ (*r* = 0.12).

**Table 2:** Pearson correlation coefficients (Weighted by square root of county population)

Variable	PM_2.5_	EA^†^	LE^††^	Median Income	Percent Smokers	Percent Obese
PM_2.5_	1	−0.22*	−0.27*	0.12*	0.20*	0.27*
Exceptional Aging	−0.22*	1	0.51*	0.38*	−0.32*	−0.41*
Life Expectancy	−0.27*	0.51*	1	0.68*	−0.63*	−0.77*
Median Income	0.12*	0.38*	0.68*	1	−0.54*	−0.58*
Percent Smokers	0.20*	−0.32*	−0.63*	−0.54*	1	0.64*
Percent Obese	0.27*	−0.41*	−0.77*	−0.58*	0.64*	1

*p < 0.001

† Exceptional Aging

†† Life Expectancy

Table 3 presents regression results from four linear regressions (unadjusted and full models for both EA and LE). Unadjusted models yielded coefficients for income and PM_2.5_ that were larger in absolute value than coefficients from full models. For EA, the coefficients on income and PM_2.5_ were 27.39 and −33.68 respectively in the unadjusted model, compared to 12.32 and −20.22 respectively in the full model. For LE, the coefficients on income and PM_2.5_ were 0.14 and −0.12 respectively in the unadjusted model, compared to .07 and −0.05 respectively in the full model.

**Table 3:** Linear regression results. Coefficients represent the changes in the number of exceptionally aged individuals (per 10,000) or years of life expectancy corresponding to a one-unit increase in the explanatory variables (units given in parenthesis)

	Unadjusted Models		Full Models	
Variable (Units)	EA^†^	LE^††^	EA^†^	LE^††^
PM_2.5_ (μg/m^3^)	−33.68**	−0.12**	−20.22**	−0.05**
Median Income ($1000s)	27.39**	0.14**	12.32**	0.07**
Daily Smokers (%)	–	–	−34.79**	−0.16**
Obesity Prevalence (%)	–	–	−30.27**	−0.12**
Black (%)	2.38**	−0.04**	1.27*	−0.05**
Hispanic (%)	4.58**	0.02**	−0.39	0
Median Age (Years)	−51.08**	−0.11**	−32.54**	−0.06**
Over 65 (%)	61.37**	0.17**	39.74**	0.10**
Migration Rates				
1980s, 55–60-year-olds^‡^	−7.51**	–	−8.14**	–
1980s, 60–64-year-olds^‡^	21.07**	–	21.07**	–
1990s, 65–70-year-olds^‡^	−9.21**	–	−9.92**	–
1990s, 70–74-year-olds^‡^	26.46**	–	29.19**	–
2000s, 75+ year-olds^‡^	21.64**	–	21.4**	–
Census Division Indicators^§^	Included	Included	Included	Included
R^2^	0.87	0.78	0.89	0.85
N	2,996	2,996	2,996	2,996

*p < 0.05; **p < 0.001

† Exceptional Aging (regressions weighted by square root of county population)

†† Life Expectancy (regressions weighted by inverse of life expectancy confidence intervals)

‡ Age-specific migration rates were calculated by the net migration over the given decade divided by the expected population at the end of the decade, times 100, where net migration is the observed final population minus the expected final population

§ The nine census divisions are defined as follows by the U.S. Census Bureau: New England, Middle Atlantic, East North Central, West North Central, South Atlantic, East South Central, West South Central, Mountain, Pacific

Both the unadjusted and the full regressions described above were also estimated using only those counties with median incomes greater than $40,000. Though not presented in Table 3, the estimated coefficients for income and PM_2.5_ from this model were 24.56 and −47.28, respectively, in the unadjusted EA model, and 6.55 and −30.31, respectively, in the full EA model (p-values <0.001). For LE, coefficients from these regressions for income and PM_2.5_ were 0.11 and −0.14, respectively, in the unadjusted model, and 0.05 and −0.07, respectively, in the full model (p-values <0.001).

Although estimates were smaller for full models than for unadjusted models, all models reveal statistically significant associations between the longevity measures and PM_2.5_, smoking, obesity, and pollution. Smoking and obesity have notably high impacts on EA and LE. Given the estimated coefficients, a 1 % decrease in smoking corresponds to approximately the same increase in EA as a 1.7 μg/m^3^ reduction in PM_2.5_, a $2,800 increase in median income, or a 1.1 % decrease in obesity. For LE, a 1 % decrease in smoking corresponds to approximately the same increase in LE as a 3.2 μg/m^3^ reduction in PM_2.5_, a $2,300 increase in median income, or a 1.3 % decrease in obesity (results from fully adjusted models using all observations). Note that the high R^2^ values in both unadjusted and full models (0.78 - 0.89) presented in Table 3 are in part attributable to the inclusion of variables that are structurally related to the outcome variables (median age and percent of the population over age 65). Additionally, due to the fact that the EA index was calculated using only population counts from the census, the inclusion of migration rates in the EA models also contributes to the high R^2^ values.

**Fig. 1:**
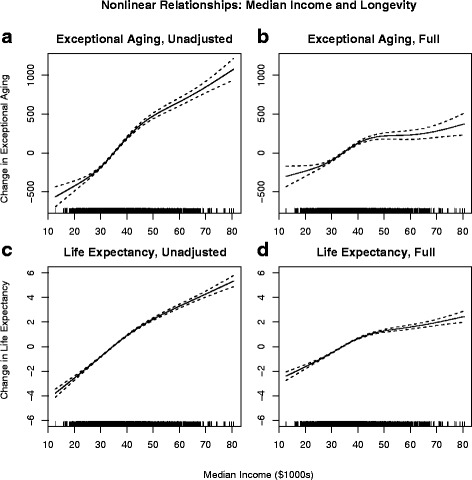
Non-linear relationships between longevity measures and median income. Panels **a** and **b** present the relationship between exceptional aging and median income for the unadjusted and fully adjusted models, respectively. Panels **c** and **d** present the relationship between life expectancy and median income for the unadjusted and fully adjusted models, respectively. Spline smooth functions allowed for up to four degrees of freedom. Unadjusted models exclude controls for smoking and obesity, while full models include these controls

Results from the linear regressions are informative, but are perhaps less valuable than the more flexible nonlinear results. Figure 1 presents estimates of the non-linear relationships between income and the two longevity measures. Panels A and C are calculated from unadjusted models and Panels B and D are from full models. These figures illustrate that the greatest gains to longevity from higher incomes occur up to about $40,000. The strength of the relationships between median income and EA and LE clearly declines with the inclusion of smoking and obesity. Moving across the entire income spectrum in the unadjusted models corresponds to an increase of almost 1,500 exceptionally aged individuals, or an 8-year increase in life expectancy. The same movement in the fully adjusted models corresponds to an increase of approximately 500 exceptionally aged individuals or a 5-year increase in life expectancy. Additionally, in the full models the pronounced bends around $40,000 illustrate that for both EA and LE, once smoking and obesity have been appropriately controlled for, marginal increases in income above $40,000 appear less valuable.

**Fig. 2:**
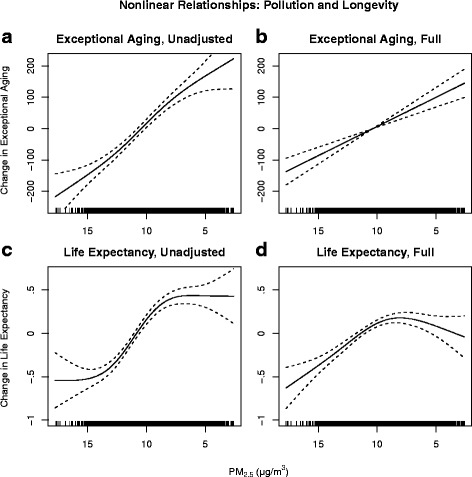
Non-linear relationships between longevity measures and PM_2.5_. Panels **a** and **b** present the relationship between exceptional aging and PM_2.5_ for the unadjusted and fully adjusted models, respectively. Panels **c** and **d** present the relationship between life expectancy and PM_2.5_ for the unadjusted and fully adjusted models, respectively. Spline smooth functions allowed for up to four degrees of freedom. Unadjusted models exclude controls for smoking and obesity, while full models include these controls

Figure 2 is analogous to Fig. 1, but presents the non-linear relationships between PM_2.5_ and the two longevity measures. Again, Panels A and C are from unadjusted models and Panels B and D are from full models. These models indicate nearly constant EA benefits from reduced air pollution at all levels of PM_2.5_. For LE the benefits appear largest when PM_2.5_ is reduced from ≈ 15 μg/m^3^ to ≈ 8 μg/m^3^. The strength of the relationships between PM_2.5_ and the longevity measures declines with the inclusion of smoking and obesity, though not as notably as for income. Moving across the entire PM_2.5_ spectrum in the unadjusted models corresponds to an increase of over 400 exceptionally aged individuals, or an approximately 1-year increase in life expectancy. The same reduction in PM_2.5_ in the full models corresponds to an increase of less than 300 exceptionally aged individuals, or an approximately 0.75-year increase in life expectancy.

**Fig. 3:**
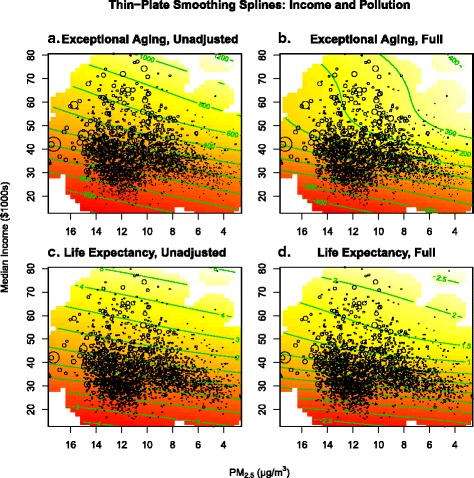
Bivariate thin-plate smoothing spline of longevity measures relative to median income and PM_2.5_. Panels **a** and **b** present the relationship between exceptional aging relative to median income and PM_2.5_ for the unadjusted and fully adjusted models, respectively. Panels **c** and **d** present the relationship between life expectancy relative to median income and PM_2.5_ for the unadjusted and fully adjusted models, respectively. Unadjusted models exclude controls for smoking and obesity, while full models include these controls

Figure 3 presents results from the thin-plate spline smoothing regressions. The green “iso-longevity” curves represent income-PM_2.5_ combinations that correspond to constant levels of longevity. Bubbles (scaled to weights) plot actual values of median income and PM_2.5_. Panels A and C of Fig. 3 are from unadjusted EA and LE models, respectively. Panels B and D come from full models. Both the values and the shapes of the iso-longevity curves change from the unadjusted to the full models.

The values of the iso-longevity curves are smaller in the unadjusted models than in the full models. In unadjusted EA models, holding PM_2.5_ constant at 10 μg/m^3^, moving from $30,000 to $40,000 corresponds to an approximately 400 individual increase in exceptionally aged individuals. In the full EA model the same change in income only corresponds to a 200 individual increase in exceptionally aged individuals. Changes in LE models are similar. In unadjusted LE models, again holding PM_2.5_ constant at 10 μg/m^3^, moving from $30,000 to $40,000 corresponds to an approximately 2-year increase in LE. In the full LE model the same increase in income only corresponds to a 1-year increase in LE.

The shapes of the iso-longevity curves also change when obesity and smoking are added to the unadjusted models. In panels A and C the widths between iso-longevity curves are fairly constant through the entire range of income, although they widen slightly for incomes above approximately $40,000. However, once smoking and obesity were included (panels B and D) the gaps between iso-longevity curves widen more quickly and dramatically for higher incomes, especially for EA. Fixing PM_2.5_ at 10 μg/m^3^, the unadjusted EA model indicates that moving from the zero iso-EA curve to the 200 iso-EA curve corresponds to a $5,000 increase in median income (≈$35 k to ≈ $40 k). In the full model, the same movement requires a $10,000 increase (≈$35 k to ≈ $45 k). This gap becomes even more pronounced when considering changes at higher levels of income. At the same fixed level of PM_2.5_, the unadjusted model indicates that to move from the 200 iso-EA curve to the 300 iso-EA curve requires a $5,000 increase in median income (≈$40 k to ≈ $45 k). In the full model, the same movement would require an increase of greater than $30,000 in median income. LE models show similar, although smaller, changes in the shapes of the iso-longevity curves.

## Discussion

The results presented 1) estimate the longevity tradeoff between income and pollution; 2) show the reduced effects of income and pollution on longevity when smoking and obesity are accounted for; 3) demonstrate that at higher levels of income, increases in income become less important relative to decreases in pollution when smoking and obesity are accounted for. Understanding these tradeoffs is particularly valuable in the case where changes in one factor are correlated with changes in other factors, as may be the case for some of the variables under consideration.

The full linear regression model provides a simple method to quantify the tradeoff between income and pollution. For EA, this model indicates that a 10 μg/m^3^ reduction in PM_2.5_ and a $16,400 increase in median income correspond to approximately the same increase in EA. The full model for LE indicates that a 10 μg/m^3^ reduction in PM_2.5_ and a $7,100 increase in median income correspond to approximately the same increase in LE. A previous study investigating tradeoffs between income and air pollution was conducted using per-capita income [21]. This study used a slightly different model (a first-difference regression model over two decades), but the results provide an interesting comparison. This study found that a 10 μg/m^3^ reduction in PM_2.5_ and a $5,000 increase (adjusted for inflation, base year 2000) in real, per-capita income corresponded to the same increase in LE. Results presented here, though not directly comparable, are similar.

Results also show that accounting for smoking and obesity significantly decreases the impact of income and pollution on longevity outcomes. This is seen not only in the linear models but also in the nonlinear figures. In Figs. 1 and 2, the decreased slopes of the lines in the full models (panels B and D) compared to the unadjusted models (panels A and C) show that the estimated longevity benefits of income and PM_2.5_ decrease once smoking and obesity have been accounted for. Additionally, the decrease in the strength of the relationship is not constant across the income spectrum. In Fig. 1, unadjusted models (panels A and C) show nearly linear relationships between income and longevity measures. However, in both panels B and D a more pronounced bend in the relationships occurs around $40,000, indicating that beyond that level increases in income are associated with smaller increases in longevity. Thus the inclusion of smoking and obesity yields non-linear results that provide some evidence of diminishing returns to income above $40,000.

In Fig. 3, this same effect is seen by observing values of the iso-longevity curves. In the full EA model, each iso-EA curve corresponds to an increase of 100 exceptionally aged individuals, compared to an increase of 200 in the unadjusted model. Similarly, each iso-LE curve in the full LE model represents a 0.5-year increase in LE whereas in the unadjusted models each iso-LE curve represents a 1-year increase. These illustrations provide evidence that smoking and obesity may be two influential mediating factors between income and longevity.

Additionally, results demonstrate that at higher levels of median income, incremental increases in income may be less effective in improving longevity than incremental reductions in air pollution. Specifically, controlling for obesity and smoking reduces the estimated benefit of higher incomes relative to lower levels of PM_2.5_. This result is partially seen in the linear regression models, but more clearly demonstrated by the nonlinear models.

Comparisons between the unadjusted and full linear models for EA indicate that the benefit of income relative to pollution decreases with the inclusion of smoking and obesity. For LE, linear models estimated with all observations indicate the opposite: the effect of adjustment for smoking and obesity is slightly larger for PM_2.5_ than for median income. However, when considering higher levels of income this is no longer the case. In the regression which used only counties whose median incomes were higher than $40,000 it becomes more apparent that at higher levels of income LE models behave similar to EA models: the LE benefit of income relative to PM_2.5_ decreases with the inclusion of obesity and smoking.

These findings demonstrate why the thin-plate spline smoothers are much more illuminating. Similar to the linear models, they show the reduced relative benefit of higher incomes when smoking and obesity have been accounted for, but do so more comprehensively. The thin-plate spline smoothers not only allow the longevity relationships for income and PM_2.5_ to vary across the ranges of income and PM_2.5_, but also depict the impact of each factor relative to the other. In full models (panels B and D of Fig. 3), gaps between iso-longevity curves widen more quickly and dramatically as income increases. Similar to Fig. 1, iso-longevity curves become less linear at around $40,000, especially for EA. This finding that the effect of income on longevity decreases for median incomes above ≈ $40,000 could be partially explained by the fact that both obesity and smoking rates are lower on average for individuals with higher incomes [7, 8], meaning income has less of a channel through which to affect longevity.

These estimates provide insight into the tradeoff between higher incomes and lower pollution exposure. Properly controlling for smoking and obesity illustrates more clearly the tradeoff between increases in income and reductions in PM_2.5_. The increased convexity of the iso-EA curves in the full EA model (illustrated in Fig. 3, panel B) suggests that for median incomes higher than approximately $40,000 (holding smoking and obesity constant), the returns for incremental reductions in PM_2.5_ may be greater than one would expect from simply observing models that do not account for smoking and obesity. These EA results differ slightly from the LE results found in the previously discussed study [21] which reported a constant relationship between the benefits of higher income and lower air pollution. However, panels C and D of Fig. 3 illustrate that the LE results discussed here are more consistent with this previous finding.

An understanding of tradeoffs between factors that influence longevity is particularly important in situations where changes in one factor are necessarily accompanied by changes in another factor. There is some evidence that two of the relationships examined in this study fit this criteria. First, given that pollution abatement is an inherently costly endeavor, it may be the case that reductions in air pollution come at the cost of reduced incomes. The positive correlation between income and air pollution may provide some evidence for this. However, the correlation is weak, and in the present study it is only observed at the county level. Thus conclusions for any given individual cannot be drawn from this analysis. A second set of factors that may also behave this way are PM_2.5_ and obesity. Unlike the relationship between PM_2.5_ and income, desirable changes in PM_2.5_ may lead to additional desirable changes in obesity prevalence [17–19]. This result could place obesity as a mediating factor in between PM_2.5_ and the longevity measures, partially explaining the reduced estimated longevity benefit of reduced PM_2.5_ in fully adjusted models.

Tangentially, results for the different longevity measures also give insight into who stands to benefit the most from increased incomes or lower pollution. The impact of income relative to air pollution is stronger for LE ($7,100 corresponds to 10 μg/m^3^ reduction in PM_2.5_) than for EA ($16,400 corresponds to 10 μg/m^3^ reduction in PM_2.5_). Because LE rates account for deaths that occur prior to age 55, the stronger impact of income on LE may be evidence that higher incomes are less beneficial to those who have already survived to age 55. These results suggest that the tradeoff between air pollution and income may not affect everyone equally. In other words, the rich and the old may receive more benefit from reductions in air pollution than increases in income relative to the poor and the young.

This study has several strengths. Similar results are observed using two different measures of longevity, EA and LE. Another strength is that this analysis includes almost all counties in the nation. Additionally, the data that were used allow for direct controls for smoking and obesity in a recent time period, while other, older studies have excluded these measures or used proxy measurements.

The primary limitation of this study is that it uses aggregated county-level data for the analysis, with no ability to examine individual-level effects. Thus an individual seeking to improve their longevity is cautioned against drawing conclusions from this analysis, because results may be driven by a subset of the population. Because of the aggregate nature of the data, if the results were driven by such a subset, it could not be identified in this analysis. Furthermore, because of the cross-sectional nature of the analysis, all relationships discussed are correlational; references to increased incomes or decreased PM_2.5_ refer only to counties with higher incomes or lower levels of PM_2.5_ exposure, and not to trends in these variables over time. Finally, an important limitation of this study is the inability to completely account for migration in EA models. The EA index was calculated strictly from census population counts, and therefore does not allow for follow-up on individuals over time. As described elsewhere [24], the inclusion of age-specific migration rates helps to control for this issue, but cannot completely account for population mobility.

Despite these limitations, the study illustrates the tradeoff between income and air pollution, and elucidates how longevity is affected by tradeoffs between increases in income and reductions in PM_2.5_, smoking and obesity.

## Conclusion

In summary, this study demonstrates longevity tradeoffs between the benefits of higher income and lower air pollution exposure and illustrates how properly accounting for smoking and obesity, possible mechanisms through which income and PM_2.5_ may influence health, alter these tradeoffs. Studies which do not account for the countervailing effects of smoking and obesity with respect to income and air pollution may overstate the impact of income on longevity and fail to fully observe the diminishing marginal rates of substitution between increased income and decreased pollution.

## Abbreviations

EA, Exceptional aging; GAM, Generalized additive models; LE, Life expectancy; PM_2.5_, Fine particulate matter air pollution <2.5 μm in diameter
